# Modular Lidar System for Multiple Field-of-View Ranging

**DOI:** 10.3390/s24010084

**Published:** 2023-12-23

**Authors:** Luka Pogačnik, Marko Munih

**Affiliations:** Faculty of Electrical Engineering, University of Ljubljana, 1000 Ljubljana, Slovenia; marko.munih@fe.uni-lj.si

**Keywords:** lidar, FOV, redirecting, mirrors, modular, walk error, time-over-threshold

## Abstract

This paper explores the possibility of distributing the fields of view (FOVs) of a centralized lidar cluster using fixed mirrors for future use in safety applications in robotics and elsewhere. A custom modular lidar system with time-over-threshold (TOT) walk error compensation was developed for the experiments. It comprises a control board that provides the processing power and adjustable voltage regulation, and multiple individually addressable analogue front end (AFE) boards that each contain a transmitter, a receiver, time-to-digital (TDC) converters for pulse width measurements on the bot Tx and Rx side, and adjustable reference voltage generators for both the Tx and Rx pulse detection threshold. The lidar system’s performance with a target in the direct line of sight is compared to the configurations where the FOV is redirected with up to three mirrors in different configurations. The results show that the light path through the neighboring mirrors introduces a minor but noticeable measurement error on a portion of the measurement range.

## 1. Introduction

Monitoring a robot’s environment from the surface of the robot has been gaining traction in recent years. This offers insight into the robot’s surroundings for better motion planning in unstructured environments as well as improved workplace safety, offered by the more nuanced knowledge of the robot’s surroundings. Traditionally, robots were enclosed into work cells with physical barriers. Whenever a human operator would enter the work cell, robots inside it would cease operation until the operator vacated the area. Although this approach was inherently safe, it was neither space-efficient nor did it allow for any kind of human–robot collaboration (HRC). The first problem was addressed by utilizing linear lidar scanners, in accordance to the readings of which, only robots in the proximity of an operator would slow down or stop. While being a big step towards human–robot collaboration, the approach with lidar scanners still leaves a lot to be desired. For a true HRC, robots and human operators have to be able to safely move even when they are in close proximity, for which the methods mentioned above do not allow. A solution that has found its way into the industrial environment is to equip a robot with force sensors. This way, the robot can stop on collision before harming the human operator. Such robots are known as collaborative robots. Although they enable HRC, other techniques are being investigated for their benefits. In the following paragraphs, we delve into specific approaches, such as monitoring the robot’s surroundings with depth cameras or on-robot time-of-flight sensors, to highlight their potential in achieving nuanced speed and separation monitoring for enhanced human–robot collaboration.

Preventing robots and operators from moving in close proximity is enabled by speed and separation monitoring (SSM) [1]. A more nuanced SSM, where the actual distance between the robot and its surroundings is known, allows for motion planning and obstacle avoidance. One way to achieve this is to observe the work cell with depth cameras [2,3], but this approach is prone to missing details due to view occlusions, a problem alleviated by using multiple depth cameras observing the same scene from multiple perspectives [4,5]. Alternatively, the robot’s surroundings can be observed from the robots’ surface by mounting time-of-flight (ToF) sensors onto the robot. Additional benefits are derived from combining such an approach with previously mentioned stationary depth cameras [6,7]. An approach that is gaining traction is to omit the expensive depth cameras and monitor the robot’s surroundings with individual cheaper depth sensors that are distributed across the robot’s surface [8,9]. This approach suffers from the inability to detect objects in very close proximity to the sensors and, as a result, has to be augmented with additional close proximity sensors, such as capacitive, tactile sensors [10,11]. Because sensors with overlapping monitored areas may interfere, the sensor position and quantity has to be considered to minimize blind spots while maximizing the measurement rate as well [12]. Readings from on-robot depth sensors may be used for implementing a more nuanced SSM, or even to aid in obstacle avoidance and path planning [13,14]. Another thing to consider is self-detection—a situation where the ToF sensor detects a segment of the robot on which it is mounted. This problem can be solved by either simulating the expected measurements in an empty room or by calculating the expected values [15]. In either case, the exact mounting positions of the sensors has to be known. An alternative to precise mounting or measurement may be to integrate inertial measurement units into the sensor boards and execute a calibration procedure that locates the sensor boards based on the robot’s movement [16]. All the references cited so far have had the observed object in their direct line of sight.

Depth cameras and ToF sensors have been demonstrated to work using a mirror to redirect light. Examples include using stationary mirrors to simulate observing an object with multiple 3D cameras [17] and using mirrors, mounted onto a robot, to expand the field of view (FOV) of a scanning lidar [18,19,20]. Furthermore, we explored the effect of mirrors on the measurement accuracy and precision in our last article [21]. With this information, a method of distributing FOVs using mirrors can be proposed.

Monitoring the robot’s surroundings from multiple points of view scattered across the robot’s surface offers unique benefits when compared to monitoring the robot and its surroundings form an external point of view. Most importantly, this approach provides information about the surroundings from a plethora of perspectives, making one less susceptible to missing details when a small number of sensors become obstructed or fail. Although a number of previously mentioned studies [8,9,10,11,12] have tackled the problem in such a way, none have achieved the measurement throughput needed for use in an actual safety application. Using a centralized lidar system that can interleave measurements on individual channels for a higher measurement throughput has been identified as a possible solution. Furthermore, a way of distributing the FOVs of a centralized multi-channel lidar using stationary mirrors to mimic the effect of mounting discrete ToF sensors across the robot’s segments is proposed. As a step towards the actual implementation of such technology, this article presents the modular lidar system that was developed for the purpose of investigating the possible problems that may arise when multiple nearby FOVs are redirected using mirrors, and the results of that investigation.

Beyond Section 1, the introduction, this paper follows an organized structure: Section 2 discusses the underlying principles, measurement equipment, and the setup of the lidar system developed for the experiments, providing comprehensive details of its design. In Section 3, we present the experimental results, conveyed through the demonstration of various measurement configurations. The interpretation of these results is expounded in Section 4. Finally, Section 5 concludes the paper and suggests potential avenues for future research.

## 2. Materials and Methods

This section introduces a modular lidar system designed to meet specific needs, along with details about the measurement setup and procedures employed. It begins with a brief overview of Time-of-Flight (ToF) ranging. This provides a foundation for a detailed explanation of the design choices and construction process behind the modular lidar system.

### 2.1. Time of Flight Ranging

Measuring distance by timing how long a signal takes to travel to the target and back has been a long-known approach. It is used in sonar, radar, and lidar, which use sound, radio, and light signals, respectively. All three rely on knowing the signal propagation speed in the medium through which they are measuring. Each has its advantages and drawbacks. Lidar suffers from complex electronics but benefits from a very high spatial resolution and a relatively constant signal propagation speed in the atmosphere. The relative speed of light at 905 nm in dry air with 450 ppm of CO_2_, at standard atmospheric pressure, ranges from 0.99978816588 at 100 °C to 0.99966055326 at −40 °C [22]. This makes the speed of light effectively constant for ranging on the surface of the Earth.

**Figure 1:**
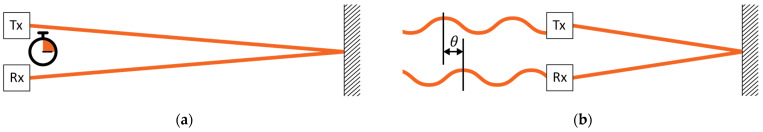
Principle of operation of (**a**) direct and (**b**) indirect time of flight measurement.

Time of flight may be measured either directly or indirectly. The former approach measures the time between transmitting a short pulse and detecting the reflection, as illustrated Figure 1a. The other method continuously modulates the transmitted light and determines the distance to the target from the phase difference between the transmitted and received signal, θ, as illustrated in Figure 1b. Direct ToF allows for greater measurement rate and may be used for detecting multiple targets, but requires more expensive hardware.

### 2.2. Modular Lidar

**Figure 2:**
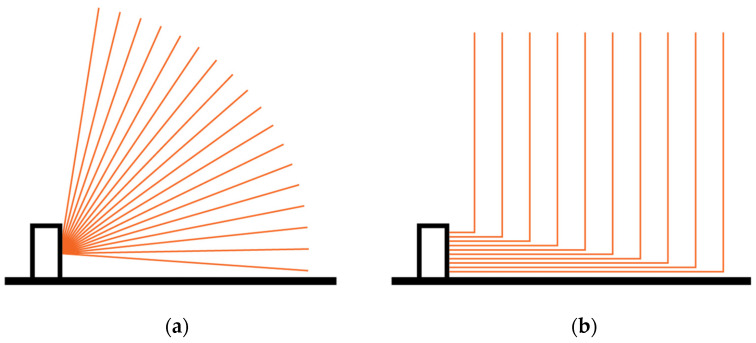
Monitoring a surface with (**a**) a scanning mirror and (**b**) statically distributed FOV lidar. The mirrors have been omitted for clarity.

The aim was to develop a lidar system that will allow us to observe the surroundings of a monitored surface, such as a robot’s segment, from the perspective of the surface. Although this has been demonstrated many times before, by using individual lidar modules [8,9,10,11,12], the aim is to attain a high enough measurement throughput to allow our solution to be usable for safety applications. Compared to monitoring the surroundings from a single point, having FOVs originate from the monitored surface directly or being redirected for the same effect is less prone to a small nearby obstacle rendering the system completely unable to observe the surroundings. That is because it is much less likely for a small obstacle to cover all FOVs when they are distributed. An example of the latter is shown in Figure 2b, while the first is illustrated in Figure 2a.

**Figure 3:**
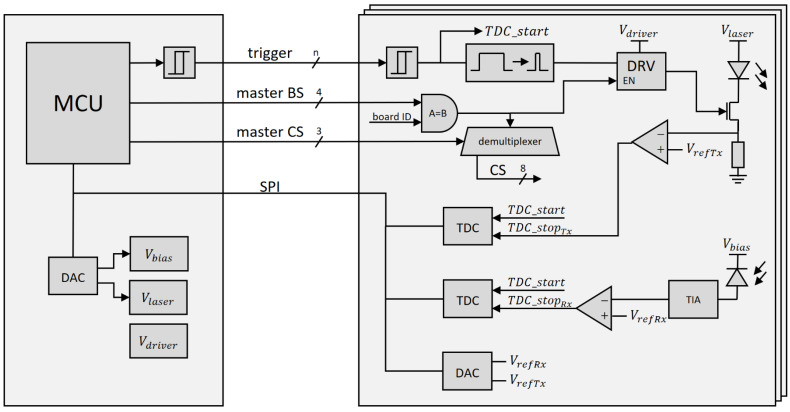
A simplified diagram of the modular lidar ranging system.

Commercially available lidar modules tend to be bulky or lack performance and desired characteristics. The most commonly used lidar modules, the Vl53Lx family from STMicroelectronics (Geneva, Switzerland), for example, offer sample rates below 100 Hz and do not allow for custom optics. Furthermore, it does not offer much insight into the reflected light properties. Building off our previously designed lidar [21], a novel direct ToF modular lidar system with a centralized processing and power supply, and up to sixteen daughter boards that each comprise a laser diode, an avalanche photodiode (APD), and a time to digital converter (TDC) has been designed. A simplified schematic is presented in Figure 3. The main board is shown on the left and the multiple daughter boards are on the right. A more detailed explanation of individual parts is explained later in this section.

#### 2.2.1. Main Board

The main board comprises a microcontroller and power supply modules. The microcontroller is powered from the USB power bus, while the remaining power modules are powered by a single 12 V external power supply. A low voltage for chips is generated using a linear voltage regulator and the remaining three voltage lines, the power for the transistor gate driver, for the laser, and for the APD, are generated using boost converters. The latter two are also digitally adjustable to allow for the fine tuning of both laser output power and APD gain. The adjustment is made using a digital to analog converter (DAC). The adjustability allows us to adjust the laser power and optimize the gain to noise ratio of the APD, which is useful for configuration but not needed during ranging operation. The main board communicates with the daughter boards over an SPI bus. Having enough distinct chip select (CS) lines would be infeasible; therefore, a four-bit master board select (BS) bus and a three-bit master CS bus is utilized. BS selects the board and master CS selects the chip on that board. The main board also controls triggering of the laser pulse. Although the design allows the trigger signal to pass through a Schmitt trigger and the connection to the daughter boards have source-terminated connections to preserve signal integrity, it was determined in testing that using a signal with a relatively slow rise time is a better approach.

#### 2.2.2. Daughter Board

**Figure 4:**
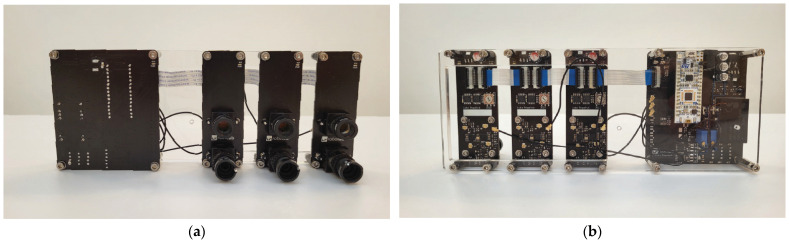
Main board (large) and three daughter boards (small) connected together (**a**) from the front, and (**b**) from the rear.

All daughter boards are connected to the main board in parallel. For all connections, with exception of the trigger signal, the daughter boards offer a pass-through connector, such that one side is connected to the main board or another daughter board, while the other side allows for further expansion, as seen in Figure 4a,b. The boards receive bias voltage for the APD, voltage for powering the laser and the transistor driver, and 5 V for powering the low-voltage chips. The latter is further decreased using a linear voltage regulator, which helps reject any noise that might be present on the 5 V line and, more importantly, eliminates problems with voltage drop, which would affect the analog functions on the daughter boards.

Up to sixteen daughter boards can be connected to a single master board. Selecting the active board is performed by matching the board’s select bus value to the desired board’s ID. This enables the MOSFET driver and the local chip select demultiplexer. With active low output, it transforms a three-bit master CS bus into individual CS signals, which control different chips. Although this allows for selecting up to eight individual chips, only five are used; one for the digital to analog converter (DAC) and four for the two TDC chips with two TDCs each.

All daughter boards’ trigger input signals are connected in parallel with separate cables. Cables are all the same length and connection points are length-matched to the common trigger on the main board. The daughter boards assume the trigger signal to be noisy, and to have a slow rise time. Therefore, the signal first passes through a Schmitt trigger to remove any noise and produce a consistent rise time. This ensures consistent delays between different components detecting the start pulse even if their input electronics exhibit slight variations. The cleaned up trigger signal is used to start both TDCs by transitioning its start signal from a low to high logic level. It simultaneously passes through a pulse shaping circuit, which generates a 50 ns pulse. If the master BS matches the board ID, the MSOFET driver is enabled, allowing the laser diode to be triggered. SPL PL90_3, a 905 nm laser diode from Osram (Munich, Germany) was used in this design. Since this laser diode does not have an integrated photodiode, the laser output power is monitored indirectly by observing the current through the diode using a shunt resistor. The signal is digitized using an analog comparator, that compares the shunt resistor voltage drop to the Tx threshold voltage, as set by the onboard DAC. The digitized pulse is timed using a TDC7201, a dual TDC from Texas Instruments (Dallas, TX, USA). Both the time to the rising edge and the time to the falling edge, corresponding to pulse start and pulse end, are recorded. This may be used to detect and correct some system variations in various working conditions which affect system delays and transistor switching times, such as change in voltage or temperature.

Measuring the reflected pulse is similar to the transmitted ones. The reflected light is gathered by an APD, MTAPD-07-013 by Marktech Optoelectronics (Latham, NY, USA). The best gain to noise ratio was found to be at 140 V reverse bias voltage. The diode’s weak output signal is amplified by an OPA857, a transimpedance amplifier from Texas Instruments with the gain set to 25 kV/A. The analog voltage is digitized using an analog comparator and the time to the rising and falling edges of the resulting signal are measured with the corresponding TDCs.

**Figure 5:**
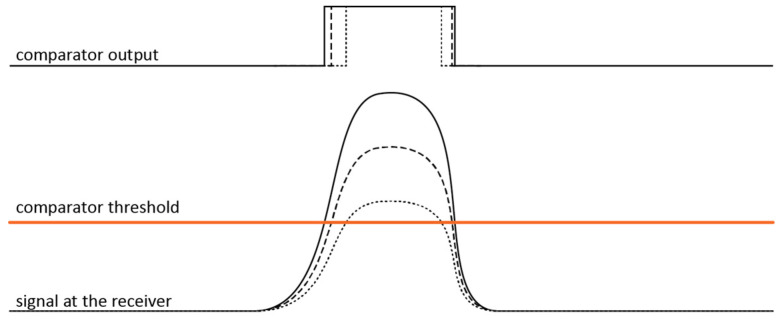
Illustration of the detection delay at different signal intensities. The solid, dashed, and dotted lines correspond to signals with different intensities where solid is the strongest, and dotted the weakest.

The delay between light pulse being transmitted and the transmission being detected with the comparator is constant, as the signal strength is only proportional to the current through the shunt resistor, which itself is only dependent on the laser voltage and various system variables, which are constant in each board. Timing the received pulse, however, is more complicated. Signal rise time is commonly defined as the time it takes for the signal to rise from 10% to 90% of its peak value, no matter the signal amplitude. On the contrary, the delay between the pulse’s onset and it passing the detection threshold strongly depends on the total signal intensity. Although a short rise time helps, alleviating the problems with timing the signal’s onset, arising from finite rise time, does not solve them entirely. This is illustrated in Figure 5, where the same pulse shape at three different intensities, and their respective comparator outputs, are shown. The signal intensity-dependent timing error is known as walk error (WE). In some cases, the error may exceed the total expected ToF.

The threshold voltages for both the transmitters and the receivers were selected through experimental adjustment. The thresholds were set as the lowest possible before noise could trigger a pulse detection.

#### 2.2.3. Walk Error Compensation

Walk error is a systemic error and may be compensated using one of many methods. Correction is commonly based on rise time [23], pulse shape [24], or time over threshold (TOT) [25]. The latter was used in our previous lidar, thus it is also used in the modular lidar system described herein.

**Figure 6:**
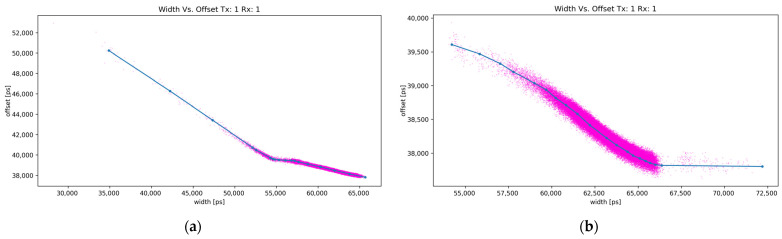
Variation in time over threshold based walk error correction with same optics but tuned for (**a**) wider FOV and (**b**) higher gain and narrower FOV. Raw values are presented with pink dots and the piece wise linear fit is presented with a blue line.

The compensation profile was obtained using a specialized setup. A spinning greyscale color wheel was mounted onto the target to provide a varying albedo. ToF and TOT were collected at multiple known distances between the lidar and the target. Walk error, defined as the difference between the measured and the expected ToF measurement, in relation to the detected pulse width, plots a continuous curve, as seen in Figure 6. This was later used to compensate the walk error of the measurements with the target at an unknown distance. The compensation profile is only good for one configuration of the transmitter, receiver, and optics. Any change in setup may significantly impact the profile of the systemic error. This is exemplified in Figure 6, where Figure 6a shows the compensation profile for when the lenses were arranged to provide a wider FOV for easier observation of reflections from neighboring transmitters, while Figure 6b shows the compensation profile for the more narrowly focused FOV. Other system parameters were kept unchanged. The compensation curves were recorded on the range of from 200 mm to 1000 mm. It is worth noting that the detected pulse width may be shorter or longer than the transmitted pulse. Figure 5 provides a graphic explanation for when the detected pulse is shorter. The signal has given rise and fall times, and lower signal amplitudes result in narrower detected pulse width. Pulse widening is attributed to the APD saturation, which leads to a delay before the signal can drop back down again [26].

When system parameters do not change, TOT-based walk error compensation works well. Problems occur when multiple reflections overlap. In that case, the apparent pulse width increases, causing the system to overcompensate, making the reading closer than in reality. This problem is similar to that of CW-modulated lidar, and similar precautions have to be taken. Most importantly, the beam of emitted light should be narrow enough such that the likelihood of it reflecting from multiple targets at substantially different distances is minimal.

#### 2.2.4. Focusing of the Optics

**Figure 7:**
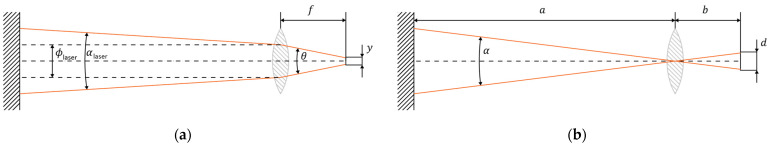
Principle of focusing light (**a**) on transmission side and (**b**) on detection side.

When selecting optics for the lidar transmitter, there are tradeoffs between beam diameter ($\phi_{laser}$) and beam divergence ($\alpha_{laser}$), which are expressed in Equations (1) and (2), respectively [27], and illustrated in Figure 7a. To obtain a narrow beam of light, the laser diode has to be placed in the lens’s focal point. In this configuration, the beam’s properties depend on source divergence (θ), source size (y), and the lens’s focal length. With a given laser diode, a shorter focal length (f) results in a narrower but more divergent beam, which may not be suitable for long-distance measurements, and a longer focal length will make the beam larger but less divergent.

(1) $$\phi_{laser}=2\cdot f\cdot \tan\left(\frac{\theta }{2}\right)+y$$

(2) $$\alpha_{laser}=2\cdot \mathrm{atan}\left(\frac{y}{2\cdot f}\right)$$

**Figure 8:**
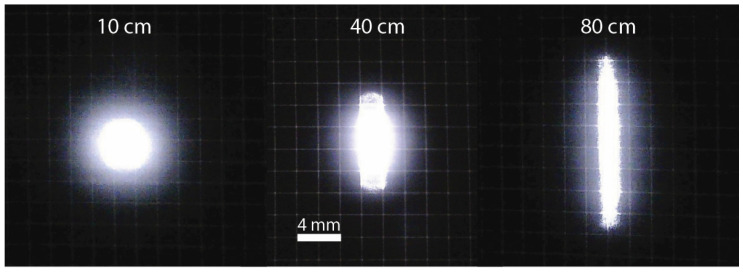
Transmitter beam shape at 10, 40, and 80 cm from the PCB on a 2 mm grid.

Another obstacle in tuning the lens is that the laser source typically is not symmetric and typically shows beam divergence and effective source locations different for directions parallel and perpendicular to the PN junction. This makes obtaining a symmetric laser spot impossible without utilizing specialized optics. The lidar system discussed in this article has had lasers focused to achieve a reasonably constant-width 2 mm beam in the horizontal direction and less than 1° in vertical divergence. The spot shape at 10, 40, and 80 cm from the PCB is shown in Figure 8. The laser spot is imaged on a black paper with white 2 mm grid. Some light bloom is present in the photos.

To obtain a sharp image with the lens, Equation (3) has to be satisfied. Here, the terms a, b, and f represent the distance between the object and lens, image plane and lens, and lens’s focal length, respectively. There is no need to obtain a sharp image when only the presence of light has to be detected.

(3) $$\frac{1}{a}+\frac{1}{b}=\frac{1}{f}$$

Even though having a sharp image on the photodiode is not necessary, having an estimate for the system’s FOV may be desirable. If the lens is focused at infinity, such that b=f, FOV (α) is calculated using Equation (4).

(4) $$\alpha =2\cdot \mathrm{atan}\left(\frac{d}{2\cdot b}\right)$$

This lidar system used M12 lens assembled with 16 mm focal length. In an idealized scenario, as described above, this would provide us with a roughly 1.8° FOV, which is good for long-range measurements at the expense of a long dead zone. The receiving optics were intentionally put out of focus such that there was the most useful signal collected on the entire measurement range. Assuming Gaussian falloff in angular sensitivity, FOV spanning with a one-sigma distribution of roughly 10° was obtained.

### 2.3. Measurement Setup

**Figure 9:**
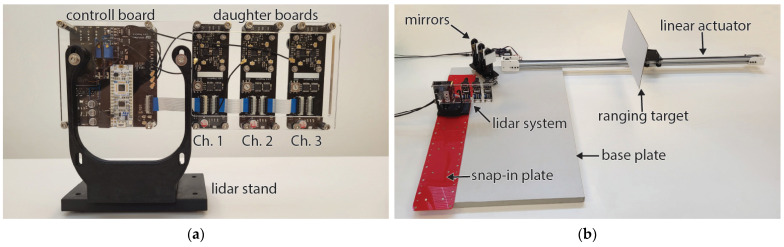
Measurement setup for (**a**) modular lidar system assembly and (**b**) ranging evaluation system for configuration mirrors. Individual components of the setups are labeled on photos.

For evaluation purposes, the modular lidar system was mounted onto a flat substrate with three individual daughterboards spaced in 40 mm increments as seen from the back side in Figure 9a. This spacing was selected following a size of the daughterboards of 30 mm wide. We allowed an additional 10 mm clearance to prevent stressing the connecting cables. Two types of configurations were evaluated. The first was direct ranging, where the lidar was positioned perpendicularly at the movable flat target such that the target was in direct line of sight. In the other configuration, the lidar system was positioned parallel to the target, such that it was not in direct FOV. Transmitted and reflected light was then redirected using mirrors, as shown in Figure 9b. Throughout each set of experiments, the mirrors were kept in fixed positions while the target moved away from them on a span of 800 mm in 10 mm increments. In each set, the same experiment was conducted with the lidar system at different distances from the mirrors in 50 mm increments. At each distance, 500 samples were collected. While collecting measurements, only one channel was transmitting light at a time, and all receivers were measuring the time to detect a reflected pulse. Results are limited to showing only ranging data for reflection gathered on the same board that transmitted the light pulse, as the others are not relevant for this study.

## 3. Results

This section contains the evaluation of the results for the modular lidar devices, first with a direct beam of light on the obstacle to be measured, followed by configurations with a light beam passing over one fixed mirror, and then combinations with two and three channels. An illustration of the light paths, based on a raytracing optical simulation, is provided at the end. For clarity, lidar channels one, two, and three correspond to the first, second, and third daughter boards of the main board, or, based on their position, as left, middle, or right, as shown in Figure 9a. The direct light beam configuration is shown with and without the walk error compensation. The configurations where the light was first redirected with a mirror include mirrors present on channels one, two, and three individually, then on all three channels simultaneously, and on pairs of channels one and two, two and three, and one and three. The results are presented in the form of graphs of the average measurement error at each specified distance between the lidar system and the fixed mirror(s).

**Figure 10:**
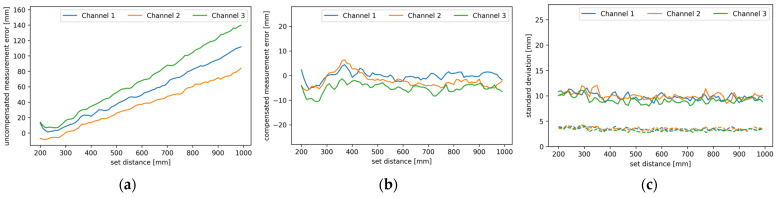
Measurement error (**a**) without walk error compensation, (**b**) with walk error compensation, and (**c**) standard deviation of walk error compensated measurements either raw (solid line) or filtered with a running average of eight samples (dashed). Different color traces represent different channels as shown in legend.

Despite the fact that the measurement results were examined for different configurations, the resulting values were found to have a standard deviation of about 12 mm over the entire calibrated measurement range, and sometimes reaching up to 15 mm beyond it, with possible algorithmic improvement, as discussed in Section 4. Measuring the direct distance to the target without walk error compensation results in an increasing positive error. This is shown in Figure 10a, where the average uncompensated measurement error is plotted against the set distance. The blue, orange, and red traces correspond to channels one, two, and three, respectively, and all the axes are in mm. Using walk error compensation, as presented in the Methods section, lowers the measurement error to that shown in Figure 10b, where the average compensated measurement error is plotted against the set distance with solid lines. As in Figure 10a, the blue, orange, and red traces correspond to channels one, two, and three, respectively, and both axes are in mm. The standard deviation of the compensated measurements was also calculated and is shown in Figure 10c. The solid lines represent the raw standard deviation of the measurements with walk error compensation, and the dashed lines represent the standard deviation of the measurements with walk error compensation after further filtering with the running average of eight samples. The standard deviation is plotted against the set distance. Again, the blue, orange, and red curves correspond to channels one, two and three, respectively; both axes are in mm.

**Figure 11:**
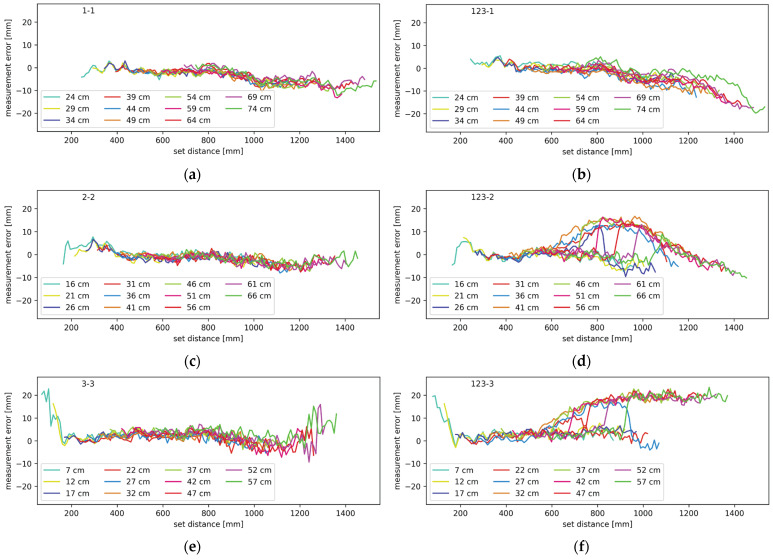
Measurement error when ranging through a mirror for (**a**) channel one with mirror only on channel one, (**b**) channel one with mirrors on all channels, (**c**) channel two with mirror only on channel two, (**d**) channel two with mirrors on all channels, (**e**) channel three with mirror only on channel three, and (**f**) channel three with mirrors on all channels.

**Figure 12:**
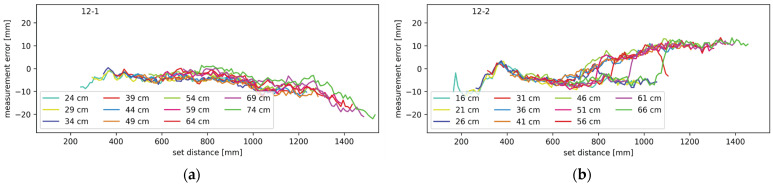
Measurement error for setup with mirrors redirecting light on channels one and two (**a**) on channel one and (**b**) on channel two.

**Figure 13:**
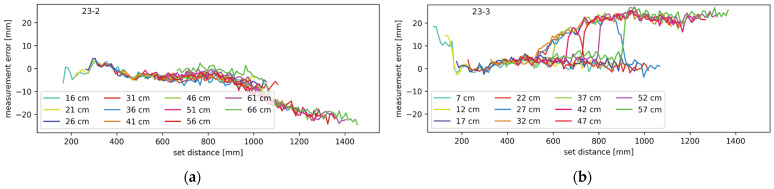
Measurement error for setup with mirrors on channels two and three (**a**) on channel two and (**b**) on channel three.

**Figure 14:**
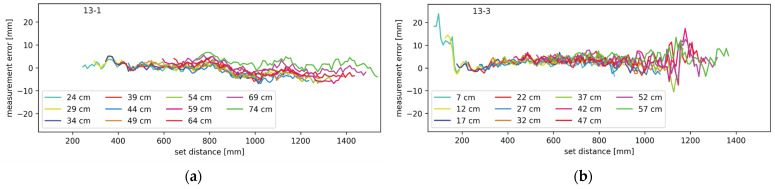
Measurement error for setup with mirrors on channels one and three (**a**) on channel one and (**b**) on channel three.

Experiments with ranging through mirrors were performed in various configurations. All combinations from one to three mirrors were tested, and the results are shown in the following diagrams. In the case of the mirror measurements, it should be noted that there are two distances involved; one from the lidar system to the mirror, and one from the mirror to the target object. The two distances combined are referred to as the set distance. Throughout the upcoming figures, each contiguous trace represents measurements from a setup where the distance between the lidar and the mirror was constant and each plot contains multiple traces at different distances between the lidar system and the mirror, as described in more detail in Section 2.3. The traces are labeled by their starting distance, which is the minimal set distance in each setup. In Figure 11, Figure 12, Figure 13 and Figure 14, the average measurement error of the walk error-compensated measurements, in mm, is plotted against the set distance, also in mm. Both the vertical and horizontal axes are kept constant throughout the figures for easier comparison. To enhance clarity, each graph is labeled in the top left corner. The digits to the left of the hyphen indicate which mirrors were present during the measurement, while the digit on the right corresponds to the lidar channel to which the plotted measurement errors belong.

The ranging performance with each channel measured through a mirror with only one mirror present is shown in Figure 11 in the left column. Diagrams (a), (c), and (e) refer to the first, second, and third channel, respectively. In this setup, only one of the three mirrors was present at a time, and it redirected only the light from the corresponding channel. This serves as a reference for what measurements through a mirror should look like without any interference. The right column of Figure 11 shows the average measurement error for the setup with all three mirrors present. Plots (b), (d), and (f) correspond to the first, second, and third channel, respectively. The plots correspond to the data where the transmitter and receiver were on the same daughter board. It can be seen that the measurement error for the setups with only one mirror was relatively constant and no significant patterns can be observed, apart from the excessive measurement error outside of the calibration range, as at the beginning in Figure 11e, and less notably, in Figure 11c. The setups with multiple mirrors, however, show notable patterns. The measurement error trends towards negative on channel one, makes positive humps on channel three and shows a combination of the two effects on channel two.

To isolate the effect of the neighboring mirrors, another experiment was conducted with only two mirrors. One was in the redirecting of the transmitted light on channel one, and the other on channel two. The results are shown Figure 12. The average measurement error of the walk error-compensated measurements is plotted against the set distance. The different colors represent the different starting distances between the lidar and the mirrors as described above. The average measurement error on channel one shows a negative trend, and positive humps are present on channel two. Those appear gradually and disappear quickly. Figure 12a,b show data for channels one and two, respectively.

Another pair of mirrors tested was a configuration with only the second and third mirror. This configuration is similar to the previous one; therefore, the results were expected to show the same trends. That is confirmed by Figure 13, which, just as before, shows the average measurement error as a function of the set distance at various starting distances. Figure 13a shows the data for channel one and Figure 13b shows the data for channel three.

There is a big similarity between the setups with mirrors present only on channels one and two, and two and three. This is confirmed by the similarity between Figure 12 and Figure 13. They illustrate the interference contribution from the mirror directly to the left or right from the monitored channel. To determine the effect of a mirror one position away from the monitored channel, a setup with mirrors present only on channels one and three was tested as well. The measurement errors for this setup on channels one and three are shown in Figure 14a,b, respectively. Again, the average measurement error is plotted against the set distance; the different traces correspond to the different minimum distances between the lidar and the mirrors. The results are very similar to those from the tests with only one mirror present.

**Figure 15:**
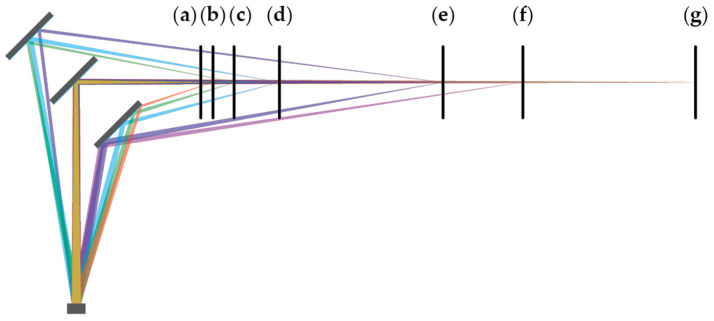
Light paths for reflected light with target at various distances from the mirror.

Some conclusions could be drawn simply by analyzing the obtained results in Figure 10, Figure 11, Figure 12, Figure 13 and Figure 14, but a more reliable explanation can be obtained by also considering a raytracing simulation [28]. A two-dimensional top-down simulation with a simplified setup with three mirrors was used. The receiver was placed where the photodiode of the middle lidar board would be, and a point light source was positioned where the target would be illuminated. This source was moved along the axis that the target was moved in the physical experiments. An illustration of the light paths from the target (vertical black line) at different distances is shown in Figure 15. It can be seen that, depending on the set distance (a–g), the reflected light is coupled to the receiver through different light paths. The light path through the central mirror, which is the one through which the light pulse is transmitted, is always present, but the light path through the left and right mirror is only present on some set distances.

In the simulation, when the target is very close to the mirrors (a), only one possible light path exists, and that is straight back through the same mirror that the light was sent through initially. With the target a little farther (b), the light can also be coupled through a mirror on the right. The reflection initially appears at the farther edge of the mirror and slowly moves towards the closer one with an increasing target’s distance. At some point (f), only partial reflection can be obtained through the mirror on the right and therefore, the contribution of this light path starts to decrease before fully disappearing (g). This light path is present on a wide range of target positions.

The light path through the mirror on the left is present on a more narrow range. Before it is partially established (c), the left mirror is occluded by the front side of the middle one. With the distance to the target increasing, this light path starts getting obstructed by the back of the mirror substrate (e) before becoming completely cut off a little farther (f). Both the slow onset of this light path and its disappearance can be seen in Figure 11, Figure 12 and Figure 13. It has to be noted that the exact distances when the different light paths become established or obstructed depend greatly on the exact mirror dimensions, spacing, laser beam size, receiving lens diameter, and the receiver active area, as well as the distance between the lidar and mirrors.

## 4. Discussion

In this section, the measurement results are summarized, and an explanation is provided for the patterns observed in the measurement error plots. The explanation is supported by evidence from the light path simulations. Before delving into these details, a comparison is made between the lidar’s ranging performance and that of the commercially available alternatives.

In direct ranging, and even when ranging through one clean mirror, the presented lidar’s ranging measurement error is safely within the ±1 cm range, and the measurement’s standard deviation was measured to be around 12 mm, as long as the set distance is within the calibrated range. That is with nothing but walk error correction. The theoretical maximum throughput of the developed lidar ranging system is 10 kHz, split among all channels, which was limited to 500 Hz due to the limitations of the current implementation. Using a faster microcontroller and better optimized code would allow for the use of the full sample rate, where averaging multiple samples or utilizing the running average would be very feasible. A running average of four samples effectively halves the measurement’s standard deviation, while using eight samples drops it safely below 4 mm, as seen in Figure 10c. According to an independent evaluation of Microsoft Kinect 2.0 [29], our lidar’s accuracy is comparable to Microsoft Kinect 2.0 but the Kinect has better precision. Since our design allows for a much higher sample rate, the precision could be matched by utilizing the running average. Based on the intended use, the presented lidar is more comparable to the Vl53L1x lidar sensor from STM, which is often used for monitoring the robot’s surroundings from the surface of the robot. Both have a similar measurement error but Vl53L1x has a slightly better measurement noise [30]. The latter could be, once again, significantly improved by utilizing the running average, which would make our sensor better than the STM’s offering.

In an ideal case, where both the field of view and field of illumination were infinitesimally narrow, and would perfectly overlap on each channel, the measurement results for the configurations with one or more mirrors should be indistinguishable from one another. Achieving such a light path is expensive to manufacture and demands a bulky optics setup. As such, differences between the configurations with one or more mirrors may be observed in some conditions. Errors that may be expected in various configurations depend on the ToF measurement technology. The device developed for the experiments in this article is a pulsed lidar with a 50 ns pulse width and time-over-threshold walk error compensation. Due to its wide pulse, it cannot discern individual reflections from separate light paths or off individual reflecting surfaces, such as the target and grime or dust on the mirrors, as individual reflections would overlap. The mirrors were thoroughly cleaned before collecting the measurements; therefore, reflections off dirt and grime are not present, as confirmed by the results for ranging with only one mirror. From this observation, it is clear that multi-path reflection is the main contributor to the measurement error.

In Figure 11, Figure 12, Figure 13 and Figure 14, certain patterns can be observed. Firstly, the mirrors only affect the ranging performance when the lidar channels are physically close to one another. Secondly, ranging is slightly affected by the mirror on the right in the form of the measurements showing the target closer than the actual distance. Thirdly, the mirror on the left notably affects the channel on the right.

Based on the measurement results and the simulations, illustrated in Figure 15, the effect of the left and right mirrors can be summarized. The right mirror contributes to an increasingly negative measurement error. Light has to travel a shorter path through the right mirror but it enters the receiving optics at a greater angle. This results in a low amplitude and slightly early reflection. Combining with the main reflection, the resulting pulse is slightly early and slightly wider than it should be. The earliness causes negative measurement error, while pulse widening causes the undercompensation of the walk error and therefore, positive measurement error. The two effects oppose each other but clearly do not cancel out. With increasing distances between the lidar and mirror, and the mirror and target, the incidence angle of the light reflecting through the mirror on the right decreases. This increases the light gain and the total amount of light that hits the receiver; thus, the error this light path brings increases as well. According to the results presented in Figure 11b,d, Figure 12a and Figure 13a, the effects of the mirror on the right become notable when the target is 80 cm from the lidar, measuring through the primary light path. The effects remain present throughout the rest of the measurement range.

The mirror on the left interferes with the measurements in slightly different way. As seen in Figure 15, the undesired light path through the neighboring mirror is longer than the primary path, making pulse widening its primary effect. This results in walk error undercompensation, which makes the measurements farther than expected. At least in some lidar and mirror configurations, this effect is only present on a narrow range. The error slowly increases in magnitude before possibly reaching a plateau and then quickly disappearing. Judging by the simulations, this error should disappear completely at some distance, even though in some of our measurements it did not. This can be attributed to the limited measurement range. With an increasing distance, the incidence angle decreases, slowly increasing both the gain and total light coupling into the receiver. At a certain angle, however, the reflected light starts being occluded by the back side of the primary mirror. Because the illuminating beam is very narrow, the distance between the reflection being slightly and fully occluded is rather small, which explains the sharp drop in the measurement error.

When the primary light path is surrounded by mirrors both on the left and on the right, the measurements are affected by both stray reflections. Both positive measurement error humps and a negative measurement error trend is present, as it can be seen in Figure 11d.

## 5. Conclusions

A modular lidar system was developed and evaluated for use in an application with multiple statically redirected FOVs. Overall, the effects of redirection using mirrors are noticeable but relatively small in magnitude. They amount to less than 3 cm of measurement error in the worst case recorded in this study. This makes the system usable for experimental purposes but not necessarily suitable for practical application. For such a use, the total error should be reduced. One way may be to use calibration akin to walk error compensation, but a more reliable approach would be miniaturization. With the mirrors spaced closer together, the distances through the separate light paths would be more alike, reducing the error. With the FOV unchanged, the light could be reflected by more mirrors, which would lead to additional sources of error. If the system were miniaturized, the transmitter and receiver optics would be closer together, which would enable a narrower receiver FOV, which would also alleviate this problem. In addition, the simulation-based optimization of the hardware layout to minimize the interference and simulation-based interference prediction could also be beneficial. The simulation of the expected errors would not only be a great enrichment for the present work, but could also help to obtain more accurate measured values. By subtracting the simulated error from the actual measured values, we could significantly improve the measurement accuracy. Miniaturization, optimization, and error prediction as well as extension to other channels are planned for future work.

## Data Availability

Data are contained within the article.
